# The Relationship Between Blood Flow and Motor Unit Firing Rates in Response to Fatiguing Exercise Post-stroke

**DOI:** 10.3389/fphys.2019.00545

**Published:** 2019-05-10

**Authors:** Spencer Murphy, Matthew Durand, Francesco Negro, Dario Farina, Sandra Hunter, Brian Schmit, David Gutterman, Allison Hyngstrom

**Affiliations:** ^1^Integrative Neural Engineering and Rehabilitation Laboratory, Department of Biomedical Engineering, Marquette University, Milwaukee, WI, United States; ^2^Department of Physical Medicine and Rehabilitation, Medical College of Wisconsin, Milwaukee, WI, United States; ^3^Department of Clinical and Experimental Sciences, Università degli studi di Brescia, Brescia, Italy; ^4^Department of Bioengineering, Imperial College London, London, United Kingdom; ^5^Department of Physical Therapy, Marquette University, Milwaukee, WI, United States; ^6^Cardiovascular Center, Medical College of Wisconsin, Milwaukee, WI, United States

**Keywords:** stroke, blood flow, motor unit, fatigue, EMG

## Abstract

We quantified the relationship between the change in post-contraction blood flow with motor unit firing rates and metrics of fatigue during intermittent, sub-maximal fatiguing contractions of the knee extensor muscles after stroke. Ten chronic stroke survivors (>1-year post-stroke) and nine controls participated. Throughout fatiguing contractions, the discharge timings of individual motor units were identified by decomposition of high-density surface EMG signals. After five consecutive contractions, a blood flow measurement through the femoral artery was obtained using an ultrasound machine and probe designed for vascular measurements. There was a greater increase of motor unit firing rates from the beginning of the fatigue protocol to the end of the fatigue protocol for the control group compared to the stroke group (14.97 ± 3.78% vs. 1.99 ± 11.90%, *p* = 0.023). While blood flow increased with fatigue for both groups (*p* = 0.003), the magnitude of post-contraction blood flow was significantly greater for the control group compared to the stroke group (*p* = 0.004). We found that despite the lower magnitude of muscle perfusion through the femoral artery in the stroke group, blood flow has a greater impact on peripheral fatigue for the control group; however, we observed a significant correlation between change in blood flow and motor unit firing rate modulation (*r*^2^ = 0.654, *p* = 0.004) during fatigue in the stroke group and not the control group (*r*^2^ = 0.024, *p* < 0.768). Taken together, this data showed a disruption between motor unit firing rates and post-contraction blood flow in the stroke group, suggesting that there may be a disruption to common inputs to both the reticular system and the corticospinal tract. This study provides novel insights in the relationship between the hyperemic response to exercise and motor unit firing behavior for post-stroke force production and may provide new approaches for recovery by improving both blood flow and muscle activation simultaneously.

## Introduction

In addition to baseline weakness, individuals with stroke can have decreased ability to generate on-going sub-maximal forces during activities, such as walking, that require repeated activation of muscles and this can limit function. For example, individuals with stroke have decreased walking endurance (Dean et al., 2001; Lloyd-Jones et al., 2009; Iosa et al., 2012), and have changes in walking kinematics and kinetics over short distances (Chen and Patten, 2008; Jonkers et al., 2009). In addition, slow walking speeds in people with stroke are associated with greater fatigability of lower limb muscles (Rybar et al., 2014). Despite the functional implications, mechanisms of neuromuscular fatigue (the acute, exercise-induced reduction in the ability to generate force) post-stroke are not well studied.

In healthy individuals and people with stroke, fatigability of muscles can be quantified as a reduction in maximal strength or power or the inability to maintain a submaximal force during ongoing contractions (Enoka and Duchateau, 2008). Fatigability of muscles can originate from factors which limit the nervous system’s ability to excite muscle tissue (central fatigue) or from factors that interfere with muscle contractile properties (peripheral fatigue) (Gandevia, 2001; Enoka and Duchateau, 2008; Hunter, 2018). Central fatigue is often quantified through estimates of voluntary activation using the superimposed twitch technique (Allen et al., 1995; Gandevia et al., 1996; Shield and Zhou, 2004). In response to fatiguing contractions, central fatigue is shown as an increase in the amplitude of a superimposed twitch (SIT) force during a maximal effort contraction. At baseline (Horstman et al., 2008) and after fatigue (Knorr et al., 2011; Bowden et al., 2014), individuals with stroke have decreased ability to voluntarily activate musculature fully even with maximal efforts. Electrophysiological studies using transcranial magnetic stimulation have shown this may be due to a loss of excitatory descending drive most likely caused by the stroke-related lesions to the motor cortex (Foltys et al., 2003; Jang et al., 2017; Peters et al., 2017). Based on studies showing decreased excitability in descending motor pathways, it is not surprising that recent evidence suggests that in people with stroke, central fatigue is a more significant contributor to fatigability of the paretic leg muscles than for the non-paretic legs and healthy controls (Riley and Bilodeau, 2002; Hu et al., 2006; Klein et al., 2010; Knorr et al., 2011; Hyngstrom et al., 2012; Bowden et al., 2014; Rybar et al., 2014; Kuhnen et al., 2015; McManus et al., 2017). These studies attribute force generating deficits to baseline reductions in cortical commands, but they do not consider the impact of the build-up of metabolic by-products on muscle function that could contribute to increased fatigability of limb muscles.

Peripheral fatigue refers to fatigue that is due to reductions in muscle contractile function. For example, excessive accumulation of metabolic by-products that occurs with muscle contractions interferes with the excitation-contraction coupling (Kent-Braun et al., 2012). Accumulation of metabolic byproducts can result from inadequate perfusion of the muscle. Following stroke, this may impact contractile properties as blood flow to the muscles of the paretic leg is decreased at rest (Ivey et al., 2004; Billinger et al., 2009; Durand et al., 2015) and during brief, submaximal contractions (Durand et al., 2015) compared with healthy controls. Moreover, limitations in the hyperemic response to exercise is related to strength and other measures of function (Durand et al., 2015). In individuals with stroke, the contribution of inadequate blood flow to decreased muscle contractile properties and fatigability of limb muscles is not known. Inadequate muscle perfusion and accumulation of metabolites within the muscle can also affect neural activation of the muscle via activation of chemo-sensitive group III and IV afferent endings in the muscle (Matthews, 1972; Martin et al., 2008). Understanding the effect of blood perfusion to exercising muscles and motor unit firing is important because cooperation between the two systems may provide a method for recovery.

The association between blood flow and individual motor unit firing behavior during intermittent fatiguing contractions post-stroke has not been studied but may provide important insight for impaired force generation and task endurance in chronic stroke. The purpose of the study was to determine the relationship between the change in blood flow with metrics of fatigue and motor unit firing behavior in stroke and controls. We hypothesized that individuals with stroke would have a blunted hyperemic response to exercise accompanied decreased motor unit firing rates and this relationship would be different than controls.

## Materials and Methods

### Participants

All participants gave informed consent before participation in this study, and the procedures were approved by the Medical College of Wisconsin Institutional Review Board (PRO190103). Ten participants with chronic, hemiparetic stroke (6 male, 4 female, 60 ± 6 years) and nine neurologically intact participants (6 male, 3 female, 63 ± 7 years) participated in the study. General inclusion criteria for stroke participants were: single, unilateral stroke (obtained through verbal communication from the physician and consistent with neurological physical examination); able to ambulate at least 30 feet (with or without an assistive device); ≥6 months post-stroke; at least 18 years old; and able to give informed consent. General exclusion criteria included for stroke participants were: brainstem stroke; any uncontrolled medical condition; contractures of any lower extremity joints; inability to follow 2–3 step commands; substance abuse; people unable to walk more than 10 feet without physical assistance; narrow angle glaucoma; chronic liver or kidney disorders; major psychiatric disorders; neurodegenerative disorders. Table 1 reports the participants’ characteristics.

**Table 1:** Participant characteristics.

Participant characteristics		
	**Control (*n* = 9)**	**Stroke (*n* = 10)**

Sex, Male	6	6
Age (yr)	60 ± 6	63 ± 7
Height (cm)	173.2 ± 14.7	172.1 ± 11.7
Weight (kg)	80.3 ± 14.6	85.9 ± 19.7
Total body fat (%)	35.8 ± 6.5	39.8 ± 4.5
Lower limb muscle mass (kg)	13.0 ± 2.6	13.0 ± 2.6
Fugl-Meyer score	NA	23 ± 7
Physical activity (Met-h/week)	14 ± 7	13 ± 7


### Torque Measurements

Participants were seated upright on a Biodex System 3 (Biodex Medical Systems, Shirley, NY, United States) with the test leg hip and knee angles at 90 degrees of flexion. Isometric knee extension torque measurements were made using a JR3 E-series load cell (JR3, Inc., Woodland, CA, United States) mounted to the dynamometer spindle by means of a custom aluminum coupling. A quarter inch aluminum arm extended from the axis of the load cell to a bracket that secured either left or right ankle attachments. The ankle attachments were secured to the test leg two inches above the lateral malleolus. The torque was acquired by an EMG-USB2+ amplifier (256-channel regular plus 16-auxiliary channels, OT Bioelettronica, Turin, Italy), low-pass filtered at 500 Hz, sampled at 2048 Hz, and recorded using the OT Biolab software. The data was then zero phased, low-pass filtered at 15 Hz using a 4th order Butterworth filter prior to analysis.

### Voluntary Activation and Resting Twitch Response

The knee extensor muscles were electrically stimulated using a constant current generator (DS7A, Digitimer, Welwyn, United Kingdom) that delivered a rectangular pulse of 100 μs duration over the quadriceps muscle group. The stimulation intensity (range 200 to 500 mA) was set at 20% above the level required to produce a maximal resting twitch amplitude that caused a supramaximal stimulation. The stimulation occurred at the peak torque (visually determined when the torque reached a steady plateau) of a maximum voluntary contraction (MVC) and is referred to as the “superimposed twitch.” Once the knee extension torque returned to 0 Nm, a second stimulation was provided to elicit the resting twitch response. The maximal torque generated by the knee extensors in response to the electrical stimulation was acquired. The superimposed twitch was calculated as the magnitude of the increase provoked by the electrical stimulus during the MVC (i.e., peak of superimposed twitch minus the peak of the MVC contraction) (Allen et al., 1995). The resting twitch was the torque magnitude generated from the electrically evoked contraction of the relaxed muscle (i.e., the magnitude of the torque produced at rest). The voluntary activation was defined here as the completeness of motor unit recruitment and firing rate during a MVC and was calculated using an interpolated twitch technique (Shield and Zhou, 2004; Klass et al., 2007):

$$Voluntary\;Activation\;(\%)=\;\left(1-\frac{superimposed\;twitch}{resting\;twitch}\right)*100\%$$

### Blood Flow Measurements

**FIGURE 1:**
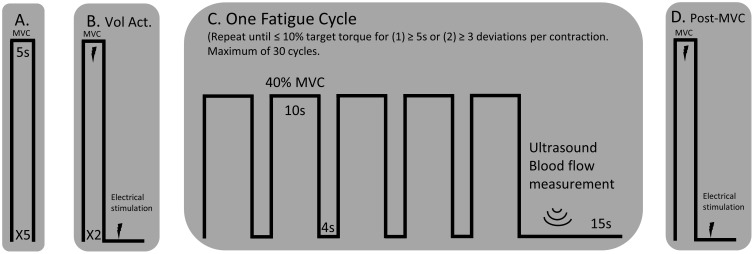
Experimental Protocol. **(A)** MVCs of the knee extensor muscles with a 1-min rest between trials. **(B)** An interpolated twitch procedure was performed for a measure of voluntary activation and resting twitch torque. **(C)** A single cycle for the fatigue protocol consisted of five consecutive 10 s isometric contractions, separated by a 4 s rest, at 40% MVC. A blood flow measurement was performed over the femoral artery of the test leg using an ultrasound machine. The fatigue cycle was repeated until the participant could not hold the isometric contraction within 10% of the target torque for more than three deviations per isometric contraction or for greater than five consecutive seconds. **(D)** A post-MVC including the interpolated twitch procedure was performed immediately after the final blood flow measurement.

The participants rested upright in the Biodex seat for a minimum of 15 min prior to assessments of blood flow. Diameter, blood flow velocity, and volume of blood flow through the superficial femoral artery were obtained with a Doppler angle of insonation fixed at 60 degrees using a linear array 4.0–12.0 MHz transducer designed for vascular imaging and equipped to a Vivid e ultrasound machine (General Electric, Fairfield, CT, United States). Sonography makes it possible to estimate the volume blood flow $\left(\frac{mL}{min}=\left(\frac{cm^{3}}{min}\right)=velocity\;\left(\frac{cm}{min}\right)\times area(cm^{2})\right)$ by careful measurement of the area, via the image, and the velocity distribution using the Doppler trace (*velocity = frequency × wavelength*) (Walter et al., 1986). Blood flow measurements during the fatigue protocol were taken immediately after each fatigue cycle (1 cycle = 5 isometric contractions at 40% MVC held for 10s and separated by a 4 s rest, see Figure 1) while the participant remained seated upright and still for a 10 s video capture of the superficial femoral artery. The same portion of the artery was able to be visualized following all fatigue cycles because the participants were secured to the chair with a lap belt and the isometric contractions did not result in movement of the lower limb. Only measurements obtained during the first three complete cardiac cycles following each fatigue cycle were included for analysis because local blood flow is a tightly coupled metabolic demand. Blood flow measurements were normalized to lean muscle mass of the whole lower limb determined by the dual-energy absorptiometry (Lunar iDXA, General Electric, Fairfield, CT, United States) analysis. Normalized blood flow was not available for one of the control participants and one of the individuals with stroke because the participants declined body composition analysis.

### Surface EMG Recordings

High-density surface electromyograms (HDsEMG) were obtained using a 64 channel 2-D electrode array with 8 mm interelectrode distance (ELSCH064NM2 – 13 rows, 5 columns, OT Bioelettronica, Turin, Italy). A double-sided adhesive sticker designed for and compatible with the array was placed over the array. The holes within the adhesive sticker were filled with a conductive electrode paste (Ten20, Weaver and Company, Aurora, CO, United States). The participant’s skin was sterilized with an alcohol swab and rubbed to remove superficial dead skin. The array was placed over the belly of the vastus lateralis, midway between the patella and the greater trochanter. A reference electrode was placed over the ipsilateral lateral malleolus. Signals for each channel were differentially amplified between 1000 and 5000 V/V (participant dependent) and band-pass filtered (10–500 Hz) using the EMG-USB2+ amplifier. The signals were sampled at 2048 Hz, A/D converted to 12 bits, and acquired using the OT Biolab software throughout the duration of the fatigue protocol.

Prior to analysis, a 2nd order bandpass filter (10–500 Hz) and a notch filter (60 Hz) were applied to each channel of the HDsEMG array. The EMG root mean square (RMS) for each channel was calculated for the five consecutive, 10 s isometric contraction $\left(RMS=\sqrt{\frac{1}{N}\sum_{n=1}^{N}x_{n}^{2}},\;n=data\;point,\;N=total\;data\;points\right)$ for the first and last cycle of the fatigue protocol (described below Figure 1). For a spectral descriptor, the mean frequency of the EMG was also calculated: $f_{\mathrm{mean}}=\frac{\int_{0}^{\frac{f_{s}}{2}}f\;S(f)df}{\int_{0}^{\frac{f_{s}}{2}}S(f)df}$, where S(*f*) is the power spectral density (PSD) of the signal and *f_s_* is the sampling frequency. The EMG signal, *x*(*t*), has a Fourier transform, *X*(*f*), and a PSD [*S*(*f*) = |*X*(*f*)|^2^/*N*]. The RMS and mean frequency values of all channels were averaged together for each fatigue cycle to provide an average RMS and mean frequency of the entire high-density surface array.

### Motor Unit Decomposition

The 63 differentially amplified sEMG channels were visually examined to exclude channels that did not show EMG activity. The remaining channels were decomposed to attain instances of single motor unit action potentials by implementing a multichannel convolutive blind source separation algorithm described and validated by Negro et al (Negro et al., 2016). In summary, the decomposition algorithm discriminates between individual motor unit action potentials from multi-unit signals. This and similar approaches have been extensively validated in previous studies (Holobar and Zazul, 2007; Holobar Minetto et al., 2010; Martinez-Valdes et al., 2017; Chen et al., 2018). The motor unit spike trains are estimated from the deconvolved sources using a peak detection algorithm and K-means classification. To provide a normalized index of reliability similar to the pulse to noise ratio, a silhouette measure (SIL) was computed on each estimated source, and the source was considered of acceptable quality if SIL was greater than 0.85. SIL provides a measure of the quality of the extracted motor unit spike trains based on the relative amplitude of the deconvolved spikes compared to the baseline noise. Motor units were identified by applying the decomposition algorithm separately to the first and last cycles of the fatigue protocol. Under the assumption that motor unit action potential shape may change with endurance, motor units were not matched between the first and last cycles; however, under the assumption of stable motor unit action potential properties, this configuration provided the possibility to identify reliably the same motor units within the same fatigue cycle (Martinez-Valdes et al., 2017).

**FIGURE 2:**
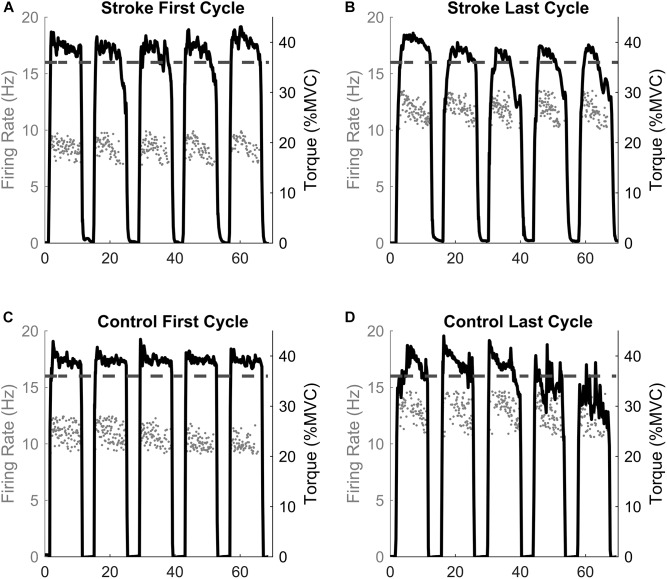
Single participant example. The motor unit firing instantaneous discharges (gray dots) and the corresponding torque generated for the first and last fatigue cycles for the stroke **(A,B)** and control **(C,D)** participants. The dashed line represents the point of task failure if the torque was not maintained above that value. Note the inability to maintain the torque above the task failure line in the last cycle compared to the first cycle.

Before calculating the discharge rates of the inter-spike intervals (ISI), abnormally long (>250 ms, 4 Hz) or short (<20 ms, 50 Hz) ISI values were excluded. The instantaneous firing rates of individual motor units were calculated as the inverse of the inter-spike interval (Hz, pulses per second, $\frac{1}{ISI}$). The motor unit action potential instances were time-locked with the torque trace. The mean firing rates were determined as the average firing rates during all five 10 s holds at 40% MVC for one fatigue cycle (i.e., First and Last Cycles, see Figure 1, 2). Therefore, the firing rate of the first fatigue cycle consists of the average firing rate for the first five contractions of the fatigue protocol, and the firing rate of the last fatigue cycle consists of the average firing rate for the last five contractions of the fatigue protocol.

### Body Composition and Clinical Measurements

Anthropomorphic measurements were performed by a licensed bio nutritionist. Body composition analysis was conducted using an iDXA (GE Lunar Medical System, Madison, WI, United States) to determine the estimated visceral fat percentage, total lower limb mass, lean muscle mass of the limbs, and percent fat composition of each limb. A Lower Extremity Fugl-Meyer (assessment of motor impairment) was performed by a licensed physical therapist.

### Experimental Protocol

The paretic leg of stroke survivors and the right leg of the control participants was tested. Figure 1 illustrates the timeline of the experimental protocol, and Figure 2 illustrates single participant examples for a control and an individual with stroke. Participants began by performing at least five baseline isometric maximum voluntary contractions (MVC) of the knee extensor muscles with 1 min of rest between trials. Visual feedback and verbal cueing were provided to the participant. The peak torque of all the trials was used as the MVC. Voluntary activation and resting twitch measures were made following the final MVC trial. A rest period of at least 5 min was provided following the final voluntary activation trial. Participants then performed the fatigue protocol. One cycle of the fatigue protocol consisted of five consecutive isometric contractions at 40% MVC and each contraction lasted for 10 s with a 4 s rest provided between each contraction. Visual feedback of the torque trace and target torque was provided using a custom written LabVIEW (National Instruments, Austin, TX, United States) program. Immediately after each fatigue cycle, a measurement of blood flow was taken. An additional 15 s of rest was applied after each cycle to allow ample time for the vascular measurements. A participant repeated the fatigue protocol until the individual was unable to hold the torque trace within 10% of the target torque for 5 s continuously or until the participant had at least three deviations of greater than 10% of the target torque within a 10 s contraction. Final vascular, voluntary activation, and resting twitch measurements were made immediately (within 10 s) following task failure. Surface EMG using the high-density electrode array was recorded throughout the duration of the fatigue protocol.

### Statistical Analysis

Data processing was performed in Matlab R2017b (Mathworks, Natick, MA, United States), and statistical tests were performed using IBM SPSS Statistics 24 (IBM, Armonk, NY, United States). Data are reported as the mean ± standard deviation. For one of the control participants and one of the individuals with stroke, body composition analysis was declined; therefore, those participants could not have blood flow normalized to lean muscle mass and were not included in that part of the analysis. The motor units for three control participants did not fulfill the SIL criteria (SIL > 0.85); therefore, only the motor units from six control participants were considered.

Separate repeated measures, mixed model ANOVAs were performed to detect statistical differences between the tested GROUP (stroke, control) and fatigue CYCLE (first and last) in: (1) normalized blood flow; (2) absolute blood flow; (3) motor unit firing rates by participant mean; (3) voluntary activation; (4) resting twitch amplitude; (5) mean EMG RMS; and (6) EMG mean frequency. The between group variable was GROUP. CYCLE was the within group comparison. A multifactorial ANOVA was used to compare the individual motor unit firing rates (main factors: 1) CYCLE (first and last fatigue cycles), and GROUP (Stroke, Control). A repeated measures ANOVA was not used for the individual motor units because detection of the same motor units at the first and last fatigue cycles could not be determined. A Bonferroni correction was used in all *post hoc* testing.

Separate one-way analysis of variances (ANOVAs) were used to test for relative change $\left[\Delta =\left(\frac{variable_{\mathrm{lastcycle}}}{variable_{\mathrm{firstcycle}}}-1\right)*100\%\right]$ in: (1) mean MVC; (2) mean EMG RMS; (3) EMG mean frequency; (4) motor unit firing rate by participant average; (5) mean blood flow (6) voluntary activation; and (7) resting twitch torque. A one-way ANOVA was also used to test for differences in task duration in terms of number of fatigue cycles completed.

Using the least squares estimates method for linear regression models, coefficient of determinations and model parameters were calculated for the following correlations: (1) Δ blood flow and task duration; (2) Δ blood flow and Δ MVC; (3) Δ blood flow and Δ motor unit firing rate; (4) absolute blood flow and MVC torque. ANOVAs were performed to test the significance of the correlations using the F-statistic. A runs test for detecting randomness was used to determine whether the linear regression fits the data. The linear regression models were included if the residuals showed a random, non-systematic pattern.

## Results

### MVC Torque and Task Duration Measurements

At baseline, controls had a greater knee extension MVC compared to individuals with stroke (129.8 ± 53.2 Nm vs. 76.4 ± 29.4 Nm, *p* = 0.003). The relative decline in MVC was similar for both groups (-39.8 ± 10.1% vs. -34.3 ± 12.6%, *p* = 0.311). There was no difference in the number of fatigue protocol cycles completed (11.9 ± 10.2 cycles vs. 15.7 ± 8.2 cycles, *p* = 0.383).

### Voluntary Activation

**FIGURE 3:**
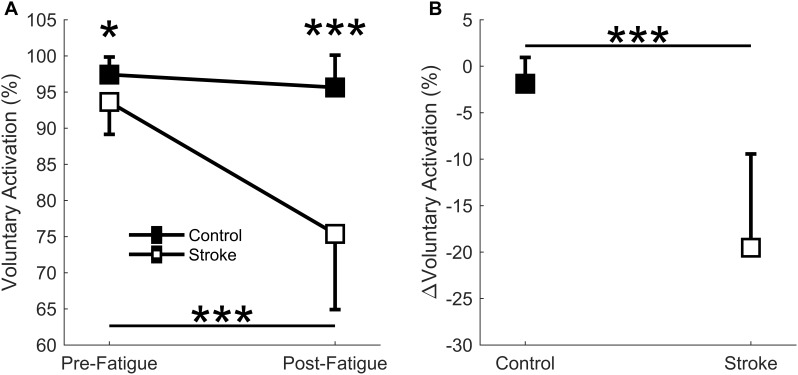
Voluntary activation. Voluntary activation assessed using the twitch interpolation technique comparing the first and last cycle **(A)** and the relative change before and after the fatigue protocol **(B)**. A significant interaction effect (*p* < 0001) was observed as individuals with stroke significantly decrease voluntary activation with fatigue. Voluntary activation was significantly lower for the stroke participants at pre-fatigue (*p* < 0.05) and post-fatigue (*p* < 0.001), and significantly decreased with fatigue for the stroke participants (*p* < 0.001) but not the controls **(A)**. A significantly greater normalized decrease in voluntary activation was observed for the stroke participants (*p* < 0.001) compared to controls **(B)**. ^∗^*p* < 0.05, ^∗∗^*p* < 0.01, and ^∗∗∗^*p* < 0.001.

A significant interaction effect was observed in the voluntary activation measurement between the control and stroke groups [*F*(1,17) = 24.9, *p* < 0.001] as the individuals with stroke had a larger decrease in the voluntary activation from the first cycle to the last cycle of the fatigue protocol (93.6 ± 4.5% to 75.4 ± 10.5, *p* < 0.001) (Figure 3A). There was no significant difference in voluntary activation of the control participants (97.4 ± 2.4% to 95.6 ± 4.5%, *p* = 0.465) with fatigue. A main effect of fatigue cycle was observed, *F*(1,17) = 37.0, *p* < 0.001, as there was a decrease in voluntary activation with fatigue. There was also a main effect of group *F*(1,17) = 24.8, *p* < 0.001, where voluntary activation in the stroke group was less than controls. Finally, the stroke group had a greater decrease in the relative change in voluntary activation compared to the controls (*p* < 0.001) (Figure 3B). In summary, the individuals with stroke had a greater decrease in voluntary activation with the fatigue protocol, as well as a lower baseline voluntary activation.

### Resting Twitch Torque

**FIGURE 4:**
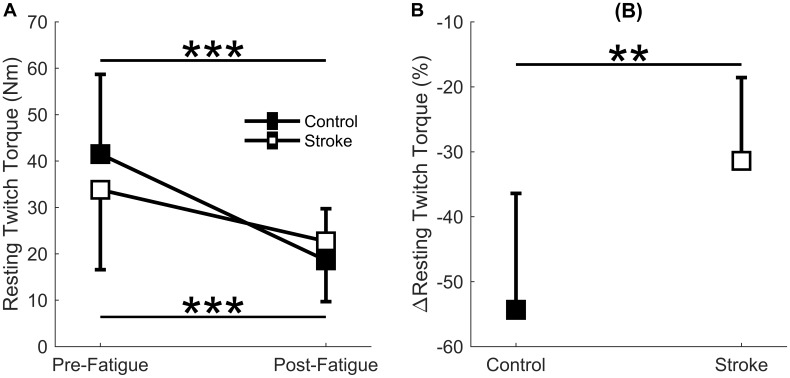
Resting twitch torque. Resting twitch torque amplitude elicited from electrical stimulation to the rectus femoris comparing the first and last cycle **(A)** and the relative change before and after the fatigue protocol **(B)**. A significant interaction effect (*p* = 0.015) was observed as control participants significantly decreased resting twitch amplitude with fatigue. Resting twitch torque significantly decreased for both the stroke participants (*p* < 0.001) and controls (*p* < 0.001) **(A)**. Controls had a significantly greater normalized decrease (*p* < 0.01) in resting twitch torque compared to the stroke participants **(B)**. ^∗^*p* < 0.05, ^∗∗^*p* < 0.01, and ^∗∗∗^*p* < 0.001.

A significant interaction effect was observed in the resting twitch torque between the control and stroke limbs [*F*(1,17) = 7.4, *p* = 0.015] as there was a greater decline in resting twitch torque response in controls (41.5 ± 17.2 Nm to 18.6 ± 11.1 Nm, *p* < 0.001) compared to individuals with stroke (33.8 ± 17.2 Nm to 22.7 ± 13.0 Nm, *p* = 0.002) (Figure 4A). There was also a main effect of cycle [*F*(1,17) = 61.3, *p* < 0.001] but not a main effect of group [*F*(1,17) = 0.8, *p* = 0.785]. Relative change in resting twitch torque showed a significantly greater decrease for the control group compared to the stroke group (*p* = 0.005) (Figure 4B). In summary, the control participants had a greater decrease in muscle contractile properties (resting twitch torque) in response to the fatigue protocol.

### Femoral Artery Blood Flow

**FIGURE 5:**
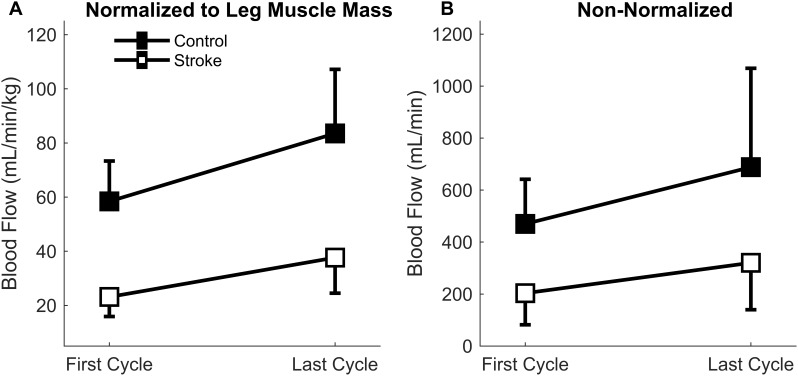
Blood Flow through the femoral artery. Blood flow normalized to leg muscle mass **(A)** and absolute magnitude **(B)** comparing the first and last cycles. Main effects of limb and fatigue cycler were observed in both cases as both groups significantly increased blood flow with fatigue and the control participants had a greater magnitude of blood flow than the individuals with stroke.

There was a significant main effect of normalized blood flow with fatigue cycle [*F*(1,15) = 24.4, *p* < 0.001] as blood flow increased in both groups with fatigue (Figure 5A). There was also a significant main effect of group [*F*(1,15) = 40.1, *p* < 0.001] as blood flow through the femoral artery was greater for the control leg compared to the paretic leg. No significant interaction effect (group^∗^cycle) was observed [*F*(1,15) = 1.7, *p* = 0.207]. Overall, lower levels of normalized blood flow were observed in the stroke group, but blood flow increased with fatigue regardless of group.

Because the normalized blood flow only applied to 17 of the participants (one control and one individual with stroke did not partake in body composition analysis), the absolute magnitude (non-normalized) was also calculated so that all participants were included (Figure 5B). Similar results were obtained for the absolute blood flow through the femoral artery in comparison to the normalized blood flow. A significant main effect of fatigue cycle [*F*(1,17) = 11.6, *p* = 0.003] and group [*F*(1,17) = 11.4, *p* = 0.004] was observed. There was no interaction effect [*F*(1,17) = 1.060, *p* = 0.318]. The data shows that regardless of group there was an increase in blood flow, but the blood flow response magnitude was greater for controls.

**FIGURE 6:**
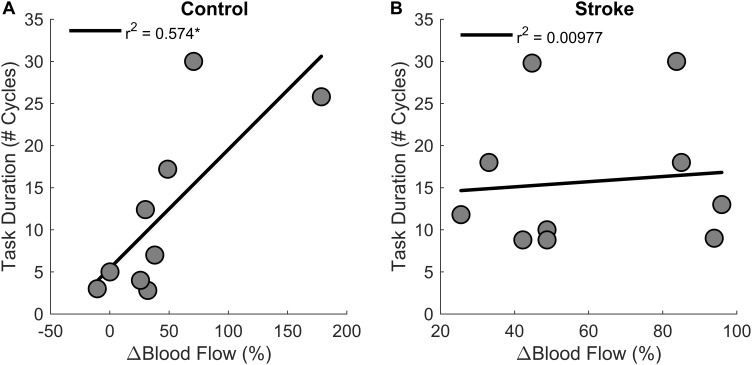
Changes in blood flow and task duration. Changes in blood flow significantly correlated with task duration for the control group (*p* = 0.018) **(A)** but not the stroke group (*p* = 0.786) **(B)**.

**FIGURE 7:**
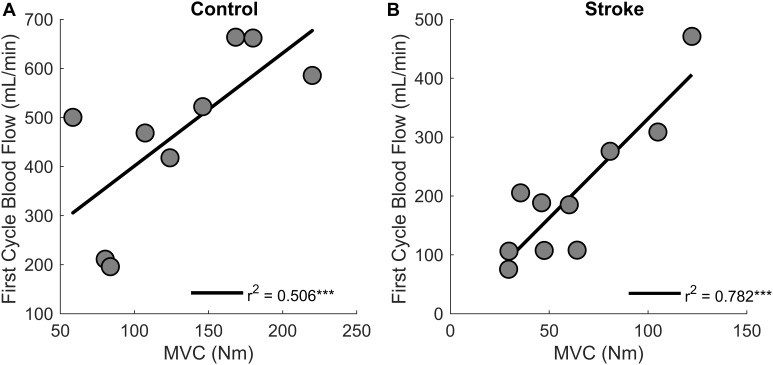
MVC and blood flow after the first cycle. Higher magnitudes of blood flow were observed for stronger individuals in both control (*p* = 0.030) **(A)** and stroke groups (*p* < 0.001) **(B)**.

Regression analysis showed that changes in blood flow significantly correlated with task duration for the control group [*r*^2^ = 0.6, *F*(1,7) = 9.4, *p* = 0.018, β = 0.141] as greater increases in blood flow related to longer task duration (Figure 6). Blood flow and task duration did not correlate for the stroke group [*r*^2^ = 0.010, *F*(1,8) = 0.079, *p* = 0.786, β = 0.031]. Blood flow after the first fatigue cycle and MVC torque generation significantly correlated together for both control [*r*^2^ = 0.51, *F*(1,7) = 7.170, *p* = 0.030, β = 2.300] and stroke participants [*r*^2^ = 0.78, *F*(1,8) = 28.6, *p* < 0.001, β = 3.375] (Figure 7), showing that stronger individuals produced larger post-contraction blood flow responses. Changes in blood flow were not significantly correlated with changes in MVC torque generation for stroke [*r*^2^ = 0.003, *F*(1,8) = 0.020, *p* = 0.890, β = -0.020] or control groups [*r*^2^ = 0.03, *F*(1,7) = 0.257, *p* = 0.628, β = 0.034].

### Motor Unit Firing Rates

**FIGURE 8:**
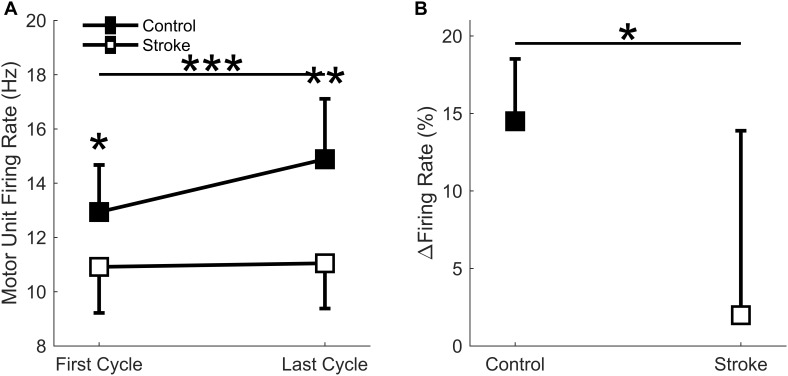
Motor unit firing rate. A significant interaction effect was observed (*p* = 0.006) as the control participants significantly increased motor unit firing rates with fatigue cycle. Stroke participants had significantly lower firing rates at the first (*p* < 0.05) and last (*p* < 0.01) fatigue cycles, and only the control group significantly increased firing rates with fatigue (*p* < 0.001) **(A)**. Control participants had a significantly greater increase in normalized firing rates compared to the stroke group (*p* < 0.05) **(B)**. ^∗^*p* < 0.05, ^∗∗^*p* < 0.01, and ^∗∗∗^*p* < 0.001.

A total of 186 motor units for the control leg (102 First Cycle, 84 Last Cycle), and 145 motor units for the participants with stroke (86 First Cycle, 59 Last Cycle) were accepted for data processing (totaling 331 motor units). When comparing the mean motor unit firing rates for the individual participants, a significant interaction effect occurred (group^∗^cycle) [*F*(1,14) = 10.3, *p* = 0.006] as there was a greater increase in firing rates for the controls as compared to stroke (Figure 8A). Main effects of group [*F*(1,14) = 10.9, *p* = 0.005] and cycle [*F*(1,14) = 13.5, *p* = 0.003] were also observed. The mean firing rates of the individual motor units for each group and cycle were: control = 13.3 ± 3.0 Hz; stroke = 10.5 ± 2.7 Hz; first cycle = 11.5 ± 3.2 Hz; last cycle = 12.8 ± 3.1 Hz. Comparisons of the mean motor unit firing rate for the first fatigue cycle showed control motor unit firing rates were significantly greater (*p* = 0.039) than the participants with stroke. The relative change in firing rate (Figure 8B) was greater for control participants (15.0 ± 3.8%) compared to the individuals with stroke (2.0 ± 11.9%) [*F*(1,14) = 6.6, *p* = 0.023]. In summary, the control leg had higher motor unit firing rates and greater motor unit discharge adaptation with fatigue compared to the individuals with stroke.

### EMG Root Mean Square (RMS)

There was a significant interaction effect (cycle^∗^group) observed [*F*(1,17) = 10.5, *p* = 0.005] as there was a greater increase in the RMS magnitude in the control participants (435.3 ± 302.3 μV to 551.9 ± 353.9 μV) as compared to individuals with stroke (244.4 ± 161.1 μV to 279.45 ± 161.1 μV). A main effect of fatigue cycle [*F*(1,17) = 36.4, *p* < 0.001] was observed, but there was not a main effect of group [*F*(1,17) = 3.9, *p* = 0.065]. The relative increase in EMG RMS was greater in the controls compared to individuals with stroke (32.4 ± 17.7% vs. 16.0 ± 9.4%), *F*(1,17) = 6.6, *p* = 0.020. In summary, the magnitude and relative change in RMS was greater for the control participants compared to the participants with stroke.

### EMG Mean Frequency

There was a main effect of fatigue cycle [*F*(1,17) = 45.6, *p* = 0.016] as the mean EMG frequency decreased (73.3 ± 14.8 Hz to 67.1 ± 14.6 Hz) from the first cycle to last cycle. There was not a main effect of group [*F*(1,17) = 0.3, *p* = 0.573], but the interaction effect (cycle^∗^group) approached significance [*F*(1,17) = 4.3, *p* = 0.054]. There was a significantly greater relative decrease in EMG mean frequency in controls compared to individuals with stroke (-14.9 ± 11.9% vs. -1.14 ± 15.6%), [*F*(1,17) = 14.5, *p* = 0.048]. In summary, the decrease in relative change and magnitude in mean EMG frequency was greater for the control than stroke participants.

### Correlations of Relative Changes in Blood Flow and Motor Unit Firing Rates

**FIGURE 9:**
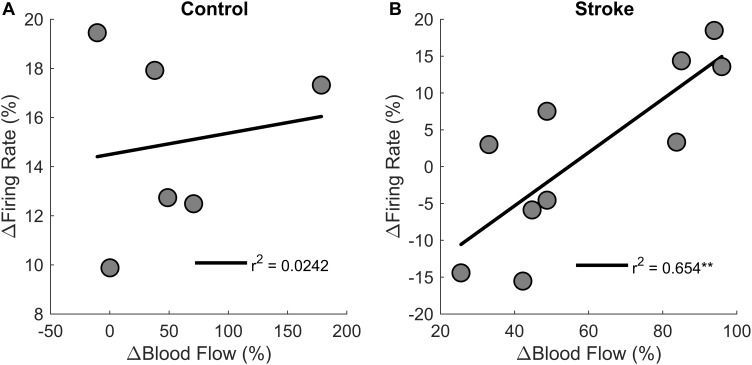
Changes in motor unit firing rates and blood flow. Change in motor unit firing rate correlated with changes in blood flow from the first fatigue cycle to the last fatigue cycle for the participants with stroke (*p* < 0.01) **(B)** but not for controls **(A)**.

Correlations were performed to test if changes in motor unit firing rates correlated with changes in blood flow from the first fatigue cycle to the last fatigue cycle (Figure 9). There was a positive correlation between change in blood flow and change in motor unit firing rates [*r*^2^ = 0.6, *F*(1,8) = 15.1, *p* = 0.004, β = 0.362] for the individuals with stroke, but not for the control group [*r*^2^ = 0.00242, *F*(1,4) = 0.1, *p* = 0.768, β = 0.009]. In summary, changes in blood flow had a significant positive correlation with changes in firing rate for the participants with stroke but not for controls, showing that motor unit firing rates correlated with blood flow only for the individuals with stroke.

## Discussion

To understand the role of muscle perfusion during fatiguing contractions after a stroke and the effect on motor unit activity, we compared the hyperemic response of healthy controls to individuals with stroke and associated this with differences in motor unit firing rates. The novel findings in this study were: (1) the net magnitude of the blood flow through the femoral artery was blunted for people with stroke compared to healthy controls during intermittent, fatiguing contractions (Figure 5); (2) greater increases in blood flow related to longer task duration for the control group but not the stroke group (Figure 6); and (3) changes in motor unit firing rates correlated with changes in blood flow for people with stroke (Figure 9). The individuals with stroke had a lower magnitude of blood flow through the femoral artery, but this did not have a lesser impact on peripheral metrics, such as resting twitch torque, than the controls; furthermore, the positive correlation among changes in motor unit firing rates and blood flow suggests a connection between muscle perfusion and neural inputs to the motoneuron. The differences seen in metrics of fatigue between controls and stroke are likely due to different mechanisms of fatigue for each group. Despite a decreased hyperemic response in the stroke group, blood flow appears to have a greater impact on peripheral fatigue and task duration for the control group. Factors which impact central fatigue such as associations between corticospinal and corticoreticular systems and inhibitory spinal reflex pathways may have a greater influence on blood flood and motor unit firing rates for the stroke group compared to the control group. Potential mechanisms and motor implications are discussed below.

### Post-stroke Differences in Hyperemia

Post-contraction hyperemia was greater for controls compared to participants with stroke (Figure 5). In addition, greater torque production resulted in greater hyperemia for both control and stroke groups (Figure 7). As was previously shown, an increased blood flow response in the paretic leg positively correlated with limb strength (Durand et al., 2015). Stronger individuals typically have a greater blood flow response, likely because of strength-associated differences in the intramuscular pressure and mechanical compression of the vessels (Barnes, 1980; Hunter et al., 2009; Durand et al., 2015). Therefore, it is likely that differences in blood flow magnitude are partially due to strength differences between and within the control and stroke groups.

It is also possible that autonomic dysregulation contributed to the blunted magnitude of the blood flow response in the individuals with stroke. After a stroke, a simultaneous decrease in parasympathetic activity and increase in sympathetic activity has been observed during Ewing’s battery assessment and during continuous monitoring of heart rate variability, blood pressure, and respiration (Dütsch et al., 2007; Xiong et al., 2014). This may increase peripheral vasoconstriction, lowering the blood flow magnitude in the stroke group compared to the control group.

### Post-stroke Blood Flow Deficits Do Not Relate to Metrics of Peripheral Fatigue or Task Duration

Healthy individuals and people with stroke had similar relative declines in MVC torque and similar time to task failure, indicating that both groups reached a similar degree of neuromuscular fatigue at task failure; however, differences in the degree of central fatigue for the stroke group compared to the degree of peripheral fatigue for the control group showed different mechanisms of fatigue. The differences in central and peripheral fatigue, as well as differences in target torque, may explain why control participants did not have longer task duration compared to the stroke participants as we expected. The control group had greater fatigue within the muscle fibers (peripheral fatigue) as indicated by larger decreases in resting twitch torque (Figure 4) and greater compression of the EMG spectrum compared to the stroke group (De Luca, 1984; Merletti et al., 1990; Riley and Bilodeau, 2002; Knorr et al., 2011). Individuals with stroke, however, showed greater decreases in voluntary activation (Figure 3), less increase in EMG RMS, and less motor unit rate modulation (Figure 8) compared to the controls. These metrics indicate some combination of decreases from descending motor command and excitatory afferent inputs, as well as increases from inhibitory afferent inputs, to the motoneuron (Gandevia, 2001; Riley and Bilodeau, 2002; Knorr et al., 2011; Bowden et al., 2014; McManus et al., 2017). We also observed that greater increases in blood flow correlated with longer task duration only for the control group (Figure 6). Thus, the differences in metrics of central and peripheral fatigue may explain why blood flow did not have a greater disturbance to muscle contractile properties for the participants with stroke.

Controls had a much greater reduction in metrics of peripheral fatigue. Although blood flow increased for both groups, changes in blood flow related to task duration only for the control group. The accumulation of metabolic byproducts, such as extracellular K^+^/Ca^2+^ from a slowed sarcoplasmic reticulum uptake and H^+^ from lactic acid (Fitts, 2011; Kent-Braun et al., 2012), decreases muscle contractile properties and conduction velocity (Tesch et al., 1983; Kupa et al., 1995). This was reflected in lower post-fatigue twitch amplitudes (Kent-Braun et al., 2012) (Figure 4) and compression of the EMG power spectrum (De Luca, 1984) for the control group but not the stroke group. This aligned with previous fatigue studies that involved sustained contractions; however, those studies did not associate these metrics with metabolite accumulation and perfusion (McComas et al., 1973; Riley and Bilodeau, 2002; Fimland et al., 2011; Knorr et al., 2011; Bowden et al., 2014; McManus et al., 2017). We were able to study the effects of blood flow on muscle fiber properties because the intermittent task (as opposed to a sustained task) involves a perfusion-dependent mechanism – greater perfusion results in reduced metabolic accumulation (Sadamoto et al., 1983). This was evident for the controls as participants with greater blood flow had longer task duration (Figure 6). The differing effects of blood flow on metrics of peripheral fatigue and task duration suggest that the influence of post-contraction hyperemia was not the same for controls and participants with stroke.

It appears the individuals with stroke were inhibited by central fatigue before blood flow was a factor in muscle force generation. One might expect the greater perfusion to result in a greater clearance of metabolites (Sadamoto et al., 1983) and greater fatigue resistance; however, blood flow did not correlate with task duration for the stroke participants (Figure 6B). Like previous studies, voluntary activation was significantly reduced at task failure (Figure 3; Newham et al., 1995; Riley and Bilodeau, 2002; Knorr et al., 2011; Bowden et al., 2014). The decreased voluntary activation assumes a decreased neural drive to the muscle (Allen et al., 1995; Shield and Zhou, 2004). This is likely facilitated by a loss of excitatory drive from the descending motor pathways after the stroke lesion ( Murase et al., 2004; Jang et al., 2017). The reduced voluntary activation not only reflects a reduction in the integrity of the corticospinal tract, but also explains a portion of the post-stroke impairments in the firing rate magnitude and rate modulation (Figure 8) manifested in the reduced EMG (Gemperline et al., 1995). It is likely that the effects of deficient blood flow on muscle fiber properties and task duration were not observed because of the dominant central factors contributing to neuromuscular fatigue.

### Motor Unit Firing Rates Correlated With Blood Flow Changes After Stroke

We saw that changes in blood flow correlated with motor unit firing rate modulation in the stroke group. Contrary to this study, previous studies observed either no change or decreases in motor unit firing rates with fatigue during sustained fatiguing contractions for both healthy individuals and individuals with stroke (Garland et al., 1994; Adam and De Luca, 2005; Mettler and Griffin, 2016; McManus et al., 2017); however, It has also been observed that motor unit firing rates increased with fatiguing contractions (Contessa et al., 2016). This study differed because motor unit firing rates were observed during intermittent contractions rather than sustained contractions. Motor unit firing rates increased in all control participants with fatigue; however, motor unit firing rates decreased in magnitude in 4/10 stroke participants. These four individuals also had lower changes in blood flow compared to those with greater rate modulation (Figure 9B). A possible explanation for the positive correlations observed for the stroke group but not the control group may be that the stroke lesion disrupted the common drive to the corticospinal and corticoreticular tracts (Buford and Davidson, 2004; Herbert et al., 2015; Brownstone and Chopek, 2018). Because corticoreticular projections originate from common areas as the corticospinal projections (primarily from the primary motor cortex and premotor cortical areas), disruptions of these common areas after a stroke likely have implications on autonomic control of blood flow from the reticular system and activation of the motoneuron pools through the corticospinal system. In animal models with decerebration or ischemic cortical lesions, inhibitory inputs from corticobulbar pathways to the reticular formation are lost, causing a greater relative increase in reticulospinal influence and a concurrent relative decrease in corticospinal influence (Hounsgaard et al., 1988; Herbert et al., 2015). This is thought to be the case in human stroke as there is evidence of increased activity in the reticulospinal pathways, possibly from the disinhibition of the reticular formation, and a loss of corticospinal input (Foltys et al., 2003; Kline et al., 2007; McPherson et al., 2008, 2018; Murphy et al., 2015; Jang et al., 2017). The decreased post-contraction blood flow through the femoral artery in this study may indicate increased autonomic drive, possibly caused by a disinhibited reticular drive providing an excessive sympathetic outflow. The reticular system is known to modulate autonomic outflow and is a source for the vasoconstrictor norepinephrine (Nette et al., 2006; Schwarz et al., 2015). The positive correlation between the hyperemic response and motor unit firing rate modulation is likely a result of the damage to these common pathways – controls and individuals with stroke that had less damage to these common pathways likely had greater modulation of motor unit firing rates and voluntary activation, as well as greater inhibition to the sympathetic outflow from the reticular formation.

An alternative explanation for the positive correlation between blood flow and motor unit modulation (Figure 9B) is that the decreased blood flow for the individuals with stroke may have an inhibitory effect on the motoneuron pool. Motor unit firing rates may be associated with blood flow because metabolic accumulation activates group III/IV afferent pathways, and these pathways are known to have an inhibitory effect on the motoneuron pool during fatiguing exercise (Kaufman et al., 1983; Amann et al., 2008; Amann, 2012; Taylor et al., 2016). The inhibitory effects of ischemia on motor unit output may also be enhanced because these pathways may be hyper-excitable after a stroke (Hidler and Schmit, 2004; Li, 2017). Recently, we showed a greater decrease in paretic motor unit firing rates during a sub-maximal contractions with a transient (5 min) bout of whole limb ischemia as compared to controls (Murphy et al., 2018). Therefore, the lack of motor unit modulation and inability to significantly increase EMG RMS may partly be due to enhanced inhibitory afferent input from group III/IV pathways. Though it is possible these afferents were activated in the control group, central factors were not significantly affected before peripheral fatigue was a factor. Enhanced group III/IV pathways may also explain why changes in motor unit firing rates significantly correlated with changes in blood flow for the stroke group but not the control group. Thus, it is plausible that the stroke-related deficiencies in the peripheral blood flow to the exercising muscle and a change in the excitability of the group III/IV afferent pathways may enhance the inhibitory response to ischemia, restricting motor unit firing rates and modulation.

### Implications for Motor Performance

Results from this study have important implications for motor performance and motor recovery post-stroke. The intermittent contractions, as opposed to sustained, showed the effects of perfusion on motor performance after a stroke. This provides insight into functional motor performance because many activities of daily living, such as walking, consist of intermittent contractions. Currently, stroke rehabilitation strategies consider cardiovascular fitness and limb strength as separate issues. This study shows the importance of considering the cooperation between the corticoreticular and corticospinal tracts in force generation after stroke. We showed that individuals with stroke that have greater post-contraction blood flow also have greater strength (Figure 7) and greater motor unit modulation (Figure 9). It seems logical that improving the hyperemic response to exercise would help improve fatigue resistance, but the data from this study would suggest that neural issues are the limiting factor to force generation during fatiguing exercise. It appears that improving neural function should remain the primary focus for rehabilitation strategies; furthermore, the evidence of increased reticulospinal influence together with the loss of corticospinal influence suggests that these two systems may be an undeveloped method for recovery. The loss of common pathways between the reticular system and spinal motoneurons may explain the systemic loss of both blood flow and motor unit rate modulation in some of the stroke participants. It is also possible that as neural function improves, perfusion to the muscle becomes more important. It has been shown that the hyperemic response to non-fatiguing muscle contractions has a positive relationship to limb function (Durand et al., 2015). Taken together, this data allows us to speculate that improving the neural contributions to the hyperemic response of limb muscles to exercise and motor unit rate modulation could optimize motor recovery in people with stroke.

## Ethics Statement

All participants gave informed consent before participation in this study, and the procedures were approved by the Medical College of Wisconsin Institutional Review Board (PRO190103).

## Author Contributions

All authors contributed to the study design, interpretation of data, approved to the final version of the manuscript to be published, and agreed to be accountable for all aspects of the work. SM and MD collected and processed the data.

## Conflict of Interest Statement

The authors declare that the research was conducted in the absence of any commercial or financial relationships that could be construed as a potential conflict of interest.
